# Crosslinking vs. Observation in Fellow Eyes of Keratoconus Patients

**DOI:** 10.1155/2022/4661392

**Published:** 2022-06-01

**Authors:** Gavin Li, Laura Di Meglio, Jiangxia Wang, Fasika A. Woreta, Kraig S. Bower, Vishal Jhanji, Divya Srikumaran, Uri S. Soiberman

**Affiliations:** ^1^Wilmer Eye Institute, Johns Hopkins University School of Medicine, Baltimore, MD, USA; ^2^Department of Biostatistics, Johns Hopkins University Bloomberg School of Public Health, Baltimore, MD, USA; ^3^UPMC Eye Center, University of Pittsburgh School of Medicine, Pittsburgh, PA, USA

## Abstract

**Purpose:**

To evaluate whether unilateral crosslinking (CXL) and conservative follow-up of the fellow eye is an acceptable management strategy in patients with keratoconus (KC).

**Methods:**

Seventy-nine fellow eyes of KC subjects that initially underwent unilateral CXL were included. Thirty fellow eyes ultimately received CXL (group 1) whereas 49 fellow eyes were followed (group 2). Best spectacle corrected visual acuity (BSCVA) and corneal tomographic parameters were collected in all eyes preoperatively and at the last follow-up.

**Results:**

Subjects who received CXL in the fellow eye (group 1) were younger than subjects who did not (group 2, *p*=0.026). Group 1 eyes had higher baseline K1 (*p*=0.026), K2 (*p*=0.006), Km (*p*=0.01), and Kmax (*p*=0.002) compared to group 2 eyes. Amongst the 49 naïve fellow eyes (group 2), 19 eyes showed evidence of progression. Progressing naïve eyes had higher baseline K1, K2, Km, and Kmax (*p* < 0.01); progressors also had thinner pachymetry at the pupil, apex, and thinnest point (*p* < 0.01). Baseline values of K1 ≥ 43.5 Diopter (D), K2 > 45.1D, Km > 44.3D, K_max_ > 47.9D, astigmatism > 1.4D, pachymetry at the pupil <475 *μ*m, and thinnest pachymetry <478 *μ*m were tentative predictors of progression in the naïve fellow eye.

**Conclusions:**

Unilateral CXL with vigilant follow up of the fellow eye may be an acceptable management strategy in a subset of KC eyes.

## 1. Introduction

Keratoconus (KC) is a corneal disorder that results in bilateral, progressive thinning of the cornea which leads to ectasia and visual deterioration from irregular astigmatism [1–3]. The disease typically manifests in early adulthood with corneal changes that can be markedly asymmetric between the two eyes of the same patient [4, 5]. Management of KC is dependent on disease progression, severity, and visual acuity. Rapid disease progression occurs in approximately 25% of KC subjects [3, 6].

Corneal collagen cross-linking (CXL) has proven to be effective in slowing or stopping the progression of KC, and it is approved by the United States Food and Drug Administration (FDA) for the treatment of progressive KC and postrefractive ectasia [7–9]. In a prospective, randomized, multicenter, controlled clinical trial in the U.S., Hersh et al. reported that CXL treatment decreased maximum keratometry and improved visual acuity one year after treatment with minimal adverse events [9]. Generally, postoperative complications are uncommon after CXL; however, infectious or sterile keratitis, persistent epithelial defects, scarring, corneal edema, and other untoward results have been described [10–13]. Vision threatening conditions that may occur after CXL are of particular interest as the procedure is more likely to be performed on young keratoconic eyes with good visual potential. Therefore, like every other surgical procedure, CXL should be performed when the benefits outweigh the potential risks.

Currently, there is a lack of consensus on whether CXL should be performed bilaterally in all KC patients. Some reports have suggested that prompt bilateral CXL may reduce overall public health costs [10]. However, even if one of two eyes is progressing, due to the asymmetrical nature of disease in each eye, there is unclear evidence regarding whether the fellow eye should be cross-linked on presentation versus followed for progression. Herein, we prospectively followed a cohort of KC eyes in an academic tertiary medical center to assess disease progression in unilaterally untreated KC eyes and to identify baseline clinical parameters that correlate with increased probability of disease progression.

## 2. Materials and Methods

### 2.1. Participants

Approval for this prospective observational patient registry was obtained from the Institutional Review Board at Johns Hopkins University, and the study was conducted in accordance with HIPPA rules (https://www.hhs.gov/hipaa/forprofessionals/privacy/index.html). This research adheres to the tenets of the Helsinki Declaration of 1964 and its later amendments. All subjects gave informed consent to participate in this study. A patient registry was compiled consisting of a cohort of 104 subjects with KC that initially underwent unilateral epithelium-off, using the Dresden protocol CXL, in the worse eye, between November 2016 and July 2019 as described in our previous study [11]. Of the 104 subjects, 12 eyes with laser-assisted in situ keratomileusis (LASIK)-induced ectasia and 12 subjects with a follow-up of less than six months were excluded from this analysis. One subject was excluded due to the fellow eye receiving a penetrating keratoplasty prior to CXL. Overall, we included 79 subjects ≥12 years old (mean 24.6 years, standard deviation [SD] 7.9 years) with naïve fellow eyes and a minimum follow-up of 6 months. Thirty subjects received CXL in the fellow eye due to evidence of KC progression (group 1). Forty-nine subjects did not receive CXL in the fellow eye throughout the follow-up period (group 2).

The criteria for performing CXL were as described in our previous study [11]. Briefly, these criteria consisted of progressive disease as defined by an increase in Kmax or steep keratometry (K2) based on Pentacam (OCULUS, Arlington, WA, USA) tomographical parameters of ≥1.0 *D* over ≤12 months. In cases where K2 or K_max_ increased by approximately ≥0.4 *D* over 3-4 months, or topographical dynamics were observed around the cone, CXL was also offered without additional wait time. Although using the cutoff of 0.4 *D* does not constitute a conservative approach, it may be useful in a subset of patients in an attempt to treat the disease as early as possible prior to the development of additional visual dysfunction, especially when tomographical worsening is seen in numerous parameters. Due to the higher risk of rapid progression in younger individuals [6, 14–16], subjects under 25 years of age were offered crosslinking immediately if best spectacle-corrected visual acuity (BSCVA) was <20/25 with tomographical evidence of keratoconus and there was a history of progressive decrease in visual acuity.

### 2.2. Data Collection

The following information was collected from every subject's medical record: demographic information, pre- and postoperative BSCVA, manifest refraction, and pre-and postoperative corneal tomographic parameters based on Pentacam measurements. Subjects were advised to halt contact lens wear for at least one week prior to each exam. The corneal tomographic data obtained included flat central keratometry (K1), steep central keratometry (K2), mean central keratometry (Km), front maximum keratometry (*K*_max_), pachymetry at the apex and thinnest point, astigmatism over the pupillary center, and anterior chamber and corneal volume. Modifying factors including the presence of atopic disease, duration of disease from diagnosis, family history of KC or myopia/astigmatism, patient-reported eye rubbing, patient-reported contact lens use, and clinical findings such as apical scarring, Vogt striae, Fleischer ring, and an observed cone were recorded. Snellen visual acuity was converted to logarithm of the minimum angle of resolution (log_MAR_) equivalent for statistical analysis.

### 2.3. Statistical Analysis

To compare the patient and eye characteristics between the CXL fellow eyes and the naïve fellow eyes, a two-sample *t*-test was used for age and Mann-Whitney test for disease duration. Pearson's chi-squared tests or Fisher's exact tests were used for comparing the categorical variables. Considering the nonnormal distribution of the corneal tomographic measurements, Mann–Whitney tests were used to compare the preoperative severity between the CXL group (1) and the naïve group (2), as well as the comparisons between the progressors and the nonprogressors within group 2. The nonparametric receiver operating curve (ROC) analysis was carried out to identify the tentative thresholds for separating the progressors from the nonprogressors. The statistical analysis software Stata version 16.1 was used. *p* values less than or equal to 0.05 were considered statistically significant.

## 3. Results

This study included 79 total fellow eyes of keratoconus patients who underwent epithelium-off, Dresden protocol CXL to one eye initially: 30 of them were treated with CXL in the fellow eye (group 1) and 49 remained treatment-naïve in the fellow eye (group 2). Within group 2, the follow-up duration ranged between 6 and 47 months, with most eyes (42) followed up for over 12 months and 7 eyes for 6–11 months. Patient and eye characteristics are described in Table 1. Overall, there were no differences in baseline characteristics between subjects who received CXL in the fellow eye and subjects with naïve fellow eyes in terms of family history of KC, ethnicity, eye laterality, presence of atopic disease, eye rubbing, median duration of disease from diagnosis, and contact lens wear. However, subjects with CXL fellow eyes (group 1) were younger than those in the naïve eye group (mean 22 ± 8.2 years vs. 26.1 ± 8.2 years; *p*=0.026). Also of note, there was an over-representation of males in the CXL group (1): 87% of the CXL fellow eyes were of male subjects, compared with 59% in the naïve fellow eye group (*p*=0.01).

Patients in group 1 were offered CXL based on either clinical symptoms (5/30) or tomographical data (25/30). During the follow-up period, a mean difference was noted in multiple parameters: K1 - 0.99±1.51D, K2 - 1.5±2.46, Kmax - 2.77±4.52D, pachymetry at thinnest point - -4.64±16.93 *μm*.

Comparing CXL fellow eyes (group 1) to naïve fellow eyes (group 2), CXL fellow eyes had more severe baseline corneal tomography parameters as shown in Table 2. CXL fellow eyes (group 1) had steeper baseline K1 (*p*=0.026), K2 (*p*=0.006), Km (*p*=0.01), and K_max_ (*p*=0.002). No differences in pachymetry at the thinnest point (*p*=0.13) or pachymetry at the apex (*p*=0.16) were observed.

We looked solely amongst the 49 naïve fellow eyes in group 2 to identify baseline differences in eyes that progressed versus those that remained stable. In this cohort, 19 eyes showed evidence of KC progression (progressors) and 30 eyes remained stable (nonprogessors).  Table 3 describes differences in baseline disease metrics for progressors and nonprogressors. Compared with nonprogressors, progressing eyes had steeper baseline K1 (*p*=0.005, K2 (*p* < 0.001), Km (*p* < 0.001), *K*_max_ (*p* < 0.001), and astigmatism over the pupillary center (*p* < 0.001). Progressing eyes had thinner pachymetry at the pupil (*p*=0.003), apex (*p*=0.003), and thinnest point (*p* < 0.001) compared to nonprogressors. Progressors also had lower baseline anterior chamber volume compared to nonprogressors (*p*=0.015). No significant differences were observed in corneal volume and BSCVA.

The progressors were followed up for an average of 22.79 ± 11.43 months. We noted changes in the following parameters: K1 increase 2.55 ± 5.16D, K2 increase 3.44 ± 5.5D, K_*max*_ increase 4.36 ± 4.52D, thinnest pachymetry decrease −9.37 ± 26.46 *μm*. The nonprogressors were followed up for an average of 21.53 ± 10.31 months. The following parameters changed during the follow-up period: K1 increase of 0.03 ± 0.32, K2 increase of 0.18 ± 0.4, Kmax increase of 0.37 ± 0.93, and thinnest pachymetry decrease of -2.80 ± 14.91 *μm*.

When the baseline characteristics of progressors vs. nonprogressors were evaluated within group 2 (naïve fellow eyes), we identified clinically relevant threshold values for several parameters associated with disease progression listed in Table 4. A K1 value of 43.5D and above was 78.95% sensitive and 66.67% specific for identifying progression in our cohort. Other topographic threshold values for identifying progressors were a K2 value of 45.1D and above (84.21% sensitive and 66.67% specific), a Km value of 44.3D and above (78.95% sensitive and 63.33% specific), a K_max_ value of 47.9D and above (84.21% sensitive and 76.67% specific), and astigmatism of over 1.4 *D* (94.74% sensitive and 60% specific). Similarly, a pachymetry value of below 475 *μ*m at the pupil (93.33% sensitive and 63.16% specific) and thinnest pachymetry below 478 *μ*m (80% sensitive and 73.68% specific) were predictors of progression.

## 4. Discussion

In this patient registry, 62% of the fellow eyes were treated with CXL. However, approximately 38% of all fellow eyes in our cohort showed no evidence of progression during follow-up and were therefore left untreated. Furthermore, we hypothesized that given the asymmetrical nature of the disease, risk factors for progression in the fellow eyes (usually with milder disease) may be unique. We have identified baseline tomographic characteristics of such fellow eyes with progressive disease in our cohort: fellow eyes with more severe disease at presentation, and specifically higher baseline K1, K2, Km, *K*_max_, astigmatism, lower pachymetry, and smaller anterior chamber volume, are more likely to progress. Our results are comparable to a previous study comparing KC progression before and after CXL, which found eyes that eventually progressed exhibited higher baseline K1, K2, and Km, and lower pachymetry values [17]. Using nonparametric ROC analysis, we were able to propose tentative threshold values that may be useful to clinicians in deciding whether to crosslink the fellow eye shortly after the first eye, or only to monitor for progression.

Currently, epithelium-off CXL is the only FDA-approved treatment that has been reported to slow or halt the progression of KC in its early to moderate stages, with numerous studies supporting its efficacy [7, 8, 11]. A previous study of bilateral sequential CXL versus delayed CXL demonstrated progression in 27% of the patients in the delayed group, and an economical analysis for the office visits saved showed that immediate CXL may be a less costly approach than delayed CXL [10]. While immediate bilateral CXL may be more appropriate for a public health system such as the one where this study originated from (the United Kingdom), it may not always be appropriate for the United States, where the cost of the only FDA- approved riboflavin formulation currently exceeds $3000 per eye, in addition to other treatment-related costs. However, to our knowledge, a cost-effectiveness analysis of CXL in the United States has not been performed.

In our series, 30 out of 49 naïve fellow eyes (61.2%) remained stable throughout a median follow-up period of 22 months. After accounting for the CXL fellow eyes (group 1) who were treated due to high risk for progression, 30 out of 79 eyes (∼38%) included in this study had stable disease. These results are consistent with a recent study by Meyer et al. which assessed the 5-year efficacy of CXL with untreated fellow eyes acting as controls. This study reported that 46% of fellow eye controls that were followed for at least 6 months had progressed in 1D or more and more than 50% remained stable [8]. Additionally, no statistically significant differences in Kmax or central corneal pachymetry were observed between treatment eyes and naïve fellow eye controls in a 2009 study of KC progression which performed unilateral crosslinking of the worse eye in 19 subjects [18]. Likewise, in a 2011 study where one eye in subjects with early/moderate KC was randomly selected for CXL, only three out of 24 untreated fellow eyes progressed with an increase in simulated keratometry and cone apex power by > 0.75D [19]. As these studies and ours demonstrate that keratoconus is stable in a considerable amount of eyes, performing CXL may unnecessarily contribute to higher medical costs for subjects, with repercussions on overall public health expenditure and also expose these stable eyes to unnecessary risks [20].

Furthermore, although Dresden protocol CXL is a safe procedure with a low complication rate, postoperative pain is quite common, and other complications may be sight threatening, including keratitis, persistent epithelial defects, corneal opacity, edema, and endothelial damage [21, 22]. Koller et al. observed a 7.6% incidence rate of sterile infiltrate after epi-off CXL using the Dresden protocol [23]. Kanellopoulos reported delayed epithelial healing in 9 out of 21 cases of KC treated with epithelium-off CXL [24]. Given the potential for complications, as low as it may be, these reports suggest that treatment with CXL should be reserved for eyes with disease progression. However, corneal dehydration and a reduction in central corneal thickness have also been noted during CXL, and may be associated with procedure-related stromal opacities and reduced endothelial cell count [25–29]. Therefore, variations to the CXL procedure have been proposed to minimize corneal dehydration. Other treatment variations aimed at reducing treatment time and minimizing risks include increasing riboflavin concentration, increasing ultraviolet-A irradiation power, and using multi-cycle pulsed ultraviolet-A delivery [12, 30]. In a preliminary clinical study assessing a proprietary riboflavin solution, the procedural safety profile was enhanced by using a more concentrated solution containing riboflavin 0.25% (versus 0.1%) and 1% hydroxypropyl methylcellulose, and by reducing treatment time using accelerated (9 mW/cm2, total dose 5.4 J/cm2) epithelium-off CXL [13]. All eyes re-epithelialized within the first 96 hours following surgery, and there was no postoperative reduction in endothelial cell density, further highlighting the favorable safety profile of this modified procedure. A demarcation line was observed at a mean depth of 362 +/− 50 *μ*m, which suggests that the treatment may potentially be as effective as the original Dresden protocol [31]. Another study examining the clinical results of accelerated pulsed-light CXL (15 mW, total dose 5.4 J/cm2, pulsed 1 : 1 second for 6 minutes) with dextran-free riboflavin 0.1% solution showed similar results with preserved endothelial cell density and a demarcation line at a mean depth of 280 +/− 32 *μ*m. No complications were noted [12]. Finally, in a recent study of accelerated pulsed epithelium-off CXL (15 mW/cm2, pulsed 2 : 1 second for 16 minutes, total energy 5.4 J/cm2) with dextran-free riboflavin 0.1% solution, complete reepithelialization was seen in all eyes four days after treatment and mild corneal haze that resolved without sequelae occurred in 13% of patients. Significant decreases in K1, K2, and Kmax were seen at 2 years [32].

Keratoconus tends to progress more rapidly in younger subjects and has been shown to plateau with age [6, 14, 15, 33, 34]. The Collaborative Longitudinal Evaluation of Keratoconus (CLEK) Study evaluating the natural history of keratoconus reported that a presenting age younger than 35 years is a significant predictor of a 3D or more increase in K1 [34]. The CLEK study likewise reported that fewer subjects progressed to transplantation later in life (12–20% aged 10–40 years vs. 3–8% aged >40 years) and that older age at baseline was protective against requiring corneal transplantation (OR 0.72) [35]. One possible mechanism by which KC progression slows or halts with age is the increase in natural nonenzymatic collagen cross-linking caused by exposure to ultraviolet radiation throughout life [36]. However, the decision to treat pediatric patients with keratoconus should not be taken lightly either: in a 5-year follow-up of 44 pediatric subjects age 18 and younger who had CXL in at least one eye, Or et al. reported that untreated fellow eyes showed no significant changes in BSCVA, Km, K_max_, mean pachymetry, or thinnest pachymetry [37]. Therefore, our suggested unilateral CXL strategy may be employed in both adult and pediatric KC patients, with ophthalmologists being especially vigilant of fellow-eye progression in younger patients.

Corneal topography, tomography, and pachymetry are the principal parameters used in the diagnosis and follow-up of progressive KC [5]. Numerous studies have identified baseline corneal characteristics that are associated with more severe disease and progression in these parameters [6, 14, 15, 33, 38]. In a study evaluating prognostic factors of KC progression that led to corneal transplantation, Tuft et al. found that eyes with steeper baseline K1 and K2 required a shorter time to penetrate keratoplasty [33]. Ferdi et al. described that thicker baseline pachymetry and steeper baseline Kmax were associated with disease progression over the follow-up period [15]. Choi et al. additionally reported thinnest pachymetry <350 *μ*m alongside an increase of ≥0.15D/year in Kmax, ≥0.2D/year in minimum keratometry, and ≥0.1D/year in central keratometry to significantly correlate with KC progression [6]. A recent study by Mimouni et al. found that in a prospective cohort of KC patients treated bilaterally with accelerated CXL, disease progression in one eye placed the fellow eyes at a higher risk for progression as well [39]. However, this study, as with the current literature at large, does not provide cutoff values in diagnostic parameters that can be used to screen naïve fellow eyes with KC for their likelihood of progression. Our study contributes to this gap in knowledge by suggesting tentative threshold values (Table 4) for corneal topography (K1, K2, Km, and Kmax) and pachymetry (pachymetry at the pupil and thinnest pachymetry) to be used in estimating the risk of progression in the naïve fellow eye of subjects treated with unilateral CXL. Combining these individual threshold values together may lead to a more accurate prediction of disease progression in fellow KC eyes using a larger cohort in the future.

The results of the study must be interpreted with caution due to several limitations. The follow-up time for group 2 ranged between 6 and 47 months, with 7 eyes having follow-up durations between 6 and 11 months and 42 eyes with follow-up durations of over 12 months. Subjects with shorter follow-up durations may have experienced further progression from the time of their last follow-up and artificially increased the percentage of nonprogressors we observed. Additionally, in order to increase the sensitivity for our analysis of KC progression, we utilized a definition of progression as any increase of > 1D in K1, K2, or Kmax over the total follow-up duration in naïve fellow eyes, even over a few years. The implication of this approach is that the rate of true progression with potential vision loss in our series may have been even lower, obviating the need for CXL to an even greater extent. However, we chose this approach in order to minimize the risk of vision loss from disease progression.

Finally, it is possible that once diagnosed with progressive disease in one eye, patient education to refrain from eye rubbing may have led to disease stabilization or at least a lack of progression.

## 5. Conclusions

We believe this study provides important considerations for the management of KC. Specifically, we suggest that unilateral CXL with careful observation of the fellow eye is an acceptable alternative to sequential bilateral CXL, at least in some KC patients. Our approach may prevent unnecessary healthcare expenditure and reduce the risks associated with CXL. The tentative baseline cutoff values proposed in this study may be beneficial to clinicians in identifying fellow naïve eyes at risk for progression. Eyes with greater risk of progressing should be monitored closely and strongly considered for CXL in the evidence of progression.

## Figures and Tables

**Table 1:** Patient Characteristics of crosslinked and naïve fellow eyes.

Patient characteristics	Overall (*N* = 79)	CXL fellow eye Group 1 *(N* = 30)	Naïve fellow eye Group 2 (*N* = 49)	*p* value
Mean age at baseline visit (years)	24.6 ± 7.9	22.0 ± 6.8	26.1 ± 8.2	0.026
Gender				
Male	55 (70%)	26 (87%)	29 (59%)	0.01
Female	24 (30%)	4 (13%)	20 (41%)	
Family history of keratoconus	7 (9%)	4 (13%)	3 (6%)	0.21
Ethnicity				
Caucasian	45 (57%)	20 (67%)	25 (51%)	0.46
African American	16 (20%)	4 (13%)	12 (24%)	
South Asian	4 (5%)	1 (3%)	3 (6%)	
East Asian	4 (5%)	1 (3%)	3 (6%)	
Other	10 (13%)	4 (13%)	6 (12%)	
*Eye characteristics*				
Eye				
Right	39 (49%)	16 (53%)	23 (47%)	0.58
Left	40 (51%)	14 (47%)	26 (53%)	
Atopic disease	32 (41%)	15 (50%)	17 (35%)	0.18
Eye rubbing	36 (46%)	12 (40%)	24 (49%)	1
Median duration (years) of disease from diagnosis	0.3 [.1, 2.0]	0.3 [0.1, 2.0]	0.8 [0.0, 5.0]	0.65
Contact lens				
Scleral	7 (9%)	1 (3%)	6 (12%)	0.52
Rigid gas permeable	11 (14%)	4 (13%)	7 (14%)	
Soft	12 (15%)	6 (20%)	6 (12%)	
Apical scar	6 (8%)	0 (0%)	6 (12%)	0.081
Vogt striae	7 (9%)	3 (10%)	4 (8%)	0.7
Fleischer ring	13 (16%)	4 (13%)	9 (18%)	0.76
Cone observed clinically	34 (43%)	14 (47%)	20 (41%)	0.63

Values are presented as mean ± SD, median [interquartile range] or as frequency (percentage).

**Table 2:** Baseline disease severity in crosslinked and naïve fellow eyes.

Variable	Overall (*N* = 79)	CXL fellow eye Group 1 (*N* = 30)	Naïve fellow eye Group 2 (*N* = 49)	*p* value
K1	43.8 [42.5, 46.1]	45.0 [43.2, 47.3]	43.5 [42.3, 45.0]	0.026
K2	46.3 [44.4, 50.7]	48.2 [45.5, 53.4]	45.2 [43.3, 49.9]	0.006
Km	45.2 [43.3, 48.1]	46.4 [44.2, 49.9]	44.4 [42.9, 46.8]	0.01
Front Kmax	50.5 [46.5, 57.4]	56.2 [49.4, 64.5]	47.8 [44.9, 56.1]	0.002
Thinnest pachymetry	480.5 [444.0, 509.0]	467.0 [434.0, 494.0]	484.0 [447.0, 516.0]	0.13
Pachymetry at the apex	493.0 [458.0, 521.0]	479.0 [440.0, 507.0]	497.0 [458.0, 526.0]	0.16

Values are presented as the median [interquartile range]. K1 flat keratometry, K2 steep keratometry, *Km* mean keratometry, *Front Kmax* front maximum keratometry. All keratometry readings are presented in Diopters, whereas pachymetry values are presented in *μ*m.

**Table 3:** Baseline disease metrics in progressing and nonprogressing naïve fellow eyes (Group 2).

Variable	Nonprogressors (*N* = 30)	Progressors (*N* = 19)	*p* value
K1	42.7 [41.8, 43.9]	44.8 [43.5, 55.5]	0.005
K2	44.1 [42.8, 46.0]	50.2 [45.2, 57.9]	<0.001
Km	43.3 [42.3, 45.3]	47.2 [44.3, 56.7]	<0.001
Front Kmax	45.9 [44.0, 47.8]	56.1 [48.3, 65.7]	<0.001
Astigmatism	1.3 [0.8, 1.9]	2.6 [1.9, 6.0]	<0.001
Pachymetry at the pupil	507.5 [492.0, 539.0]	462.0 [436.0, 508.0]	0.003
Pachymetry at the apex	508.0 [492.0, 542.0]	457.0 [392.0, 508.0]	0.003
Thinnest pachymetry	498.0 [481.0, 525.0]	447.0 [378.0, 480.0]	<0.001
Cornea volume	57.5 [56.1, 60.7]	56.7 [53.3, 60.8]	0.32
Chamber volume	202.5 [176.0, 228.0]	170.0 [152.0, 206.0]	0.015
LogMar	0.000 [0.000, 0.000]	0.097 [0.000, 0.301]	0.09

Values are presented as the median [interquartile range]. *Chamber volume*, anterior chamber volume; *LogMar*, logarithm of the minimum angle of resolution. All keratometry readings are presented in Diopters and pachymetry values are presented in *μ*m, and volume in mm^3^.

**Table 4:** Proposed tomographic cutoff values for KCN disease progression in naïve fellow eyes (*N* = 49).

Variable	Cutoff value	Sensitivity (%)	Specificity (%)	% Correctly Classified^*∗*^
K1	≥43.5	78.95	66.67	71.43%
K2	≥45.1	84.21	66.67	73.47%
Km	≥44.3	78.95	63.33	69.39%
Front Kmax	≥47.9	84.21	76.67	79.59%
Astigmatism	≥1.4	94.74	60.00	73.47%
Pachymetry at the pupil	<475	93.33	63.16	81.63%
Thinnest pachymetry	<478	80.00	73.68	77.55%

^*∗*^Percent of naïve fellow eyes correctly classified as progressors or nonprogressors using the predetermined cutoff values. All keratometry readings are presented in Diopters and pachymetry values are presented in *μ*m.

## Data Availability

The data used to compile this manuscript are available upon request to the corresponding author.
